# A Force-Visualized Silicone Retractor Attachable to Surgical Suction Pipes

**DOI:** 10.3390/s17040773

**Published:** 2017-04-05

**Authors:** Tetsuyou Watanabe, Toshio Koyama, Takeshi Yoneyama, Mitsutoshi Nakada

**Affiliations:** 1 Institute of Science and Engineering, Kanazawa University, Kanazawa 9201192, Japan; toshio83@stu.kanazawa-u.ac.jp (T.K.); yoneyama@se.kanazawa-u.ac.jp (T.Y.); 2 Department of Neurosurgery, Kanazawa University, Kanazawa 9208641, Japan; mnakada@med.kanazawa-u.ac.jp

**Keywords:** force sensor, retraction, suction, force visualization mechanism

## Abstract

This paper presents a force-visually-observable silicone retractor, which is an extension of a previously developed system that had the same functions of retracting, suction, and force sensing. These features provide not only high usability by reducing the number of tool changes, but also a safe choice of retracting by visualized force information. Suction is achieved by attaching the retractor to a suction pipe. The retractor has a deformable sensing component including a hole filled with a liquid. The hole is connected to an outer tube, and the liquid level displaced in proportion to the extent of deformation resulting from the retracting load. The liquid level is capable to be observed around the surgeon’s fingertips, which enhances the usability. The new hybrid structure of soft sensing and hard retracting allows the miniaturization of the retractor as well as a resolution of less than 0.05 N and a range of 0.1–0.7 N. The overall structure is made of silicone, which has the advantages of disposability, low cost, and easy sterilization/disinfection. This system was validated by conducting experiments.

## 1. Introduction

**Figure 1:**
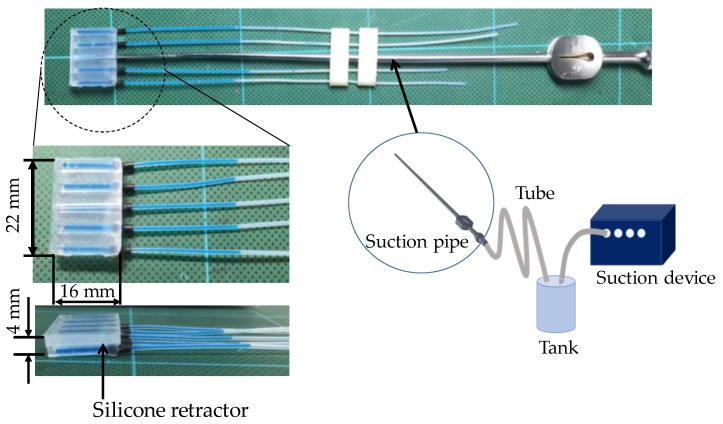
Suction pipe/device and silicone retractor for the suction pipe [1].

Retractors and suction devices are the most frequently used instruments in neurosurgery. Retractors are used to pull back tissues to produce a visible field and a space for surgeons. When retracting, an overload of retracting is a key issue. It could cause unexpected damage to brain tissues and bleeding. As the brain tissue is very soft and fragile, it can break or get damaged even with a small load. The brain is a very important organ in the human body, and any damage or injury to the brain tissue might led have serious consequences. Suction devices are mainly used to suck blood and saline for washing the brain tissue. They are also sometimes used for retracting tissues, although the retracting area is very small and limited in availability. If combining the functions of retraction and suction while avoiding the overload of retracting, surgeons can operate easily, safely, and speedily. With this in mind, we previously developed a force sensible silicone retractor that can be used by attaching it to the tip of a suction pipe [1], as shown in Figure 1. With this instrument, surgeons can perform the functions of suction, retraction, and force sensing, without changing instruments. However, the developed instrument has two drawbacks. First, available cases are limited due to its large thickness (4 mm). It was difficult to retract deep areas or to insert the instrument into narrow spaces; Second, the previously developed instrument has five independent force sensors. The distributed forces can be observed, although it is hard for surgeons to observe the separate force values simultaneously, and the space occupied by the five tubes for the sensors disturbs and reduces the usability of the instruments. With these points in mind, this paper presents an extended version of the silicone retractor that addresses these limitations. The key features of the new silicone retractor include the following:

- *Force visualization mechanism utilizing a liquid*: A liquid was installed into the sensing component of the silicone retractor. A transparent tube was connected to the sensing component to observe the liquid level, which is displaced according to the load. The load is visually measurable by the liquid displacement.
- *High usability*: The silicone retractor is easily set up by installing a suction pipe into the hole in the retractor. The retractor was composed entirely of silicone and connected to PTFE tubes. Thus, the benefits of the system include the absence of electrical components, disposability, easy sterilization/disinfection, magnetic resonance imaging (MRI) compatibility, and low cost. The sensor was installed in only one area where the largest force is exerted to minimize the damage to the brain tissue. Moreover, the fact that there is only one sensing area minimizes the space occupied by the sensor and enhances the usability of the device.
- *Multiple functions*: The proposed instrument provides the functions of retraction, suction, and force sensing via attachment to the suction pipe.
- *Hybrid soft and hard structure*: The silicone retractor must be stiff for retracting tissues, while the sensing component inside the retractor must be soft to provide high sensitivity. To realize both these functions, a hybrid soft and hard structure was adapted. A thin retractor was developed using hard retracting and soft sensing parts.

The rest of this paper is organized as follows: after describing relevant literatures, the proposed instrument and the force sensing system are presented. Several versions of the silicone retractors are then described with an experimental evaluation.

## 2. Related Work

The key function of the presented medical instrument is force sensing. Here, we focus on force sensing [2,3,4,5,6,7]. Several force sensing systems have been developed for neurosurgery. Our group developed forceps where gripping and pulling forces are measureable [8,9,10]. The group of Yang et al. developed a force-limiting dissector [11] and demonstrated its effect at real operations [12]. Interaction forces between tool and tissue were quantified by utilizing force sensors [13,14]. The used force sensors were based on strain gauges, and electrical components are needed for transferring the force information. No electrical components are preferred for easy disinfection or sterilization, which is necessary for medical operations.

Several approaches do not require electrical components for transferring the force information. A pneumatic force can be transferred via an air tube. Based on this concept, the group of Kawahara et al. [15,16] developed force sensor-embedded forceps whose tip has no electrical components. Kawahara et al. utilized air pressure with an endoscope for estimating organ stiffness [17]. Acoustic information can also be transferred from the tip to the roots of the forceps without any electrical components. Fukuda et al. utilized acoustic reflection for estimating tissue softness [18]. Optical information can be transferred via fibers without any electrical components. Peirs et al. utilized optical fibers to transfer the visualized deformation of a flexible structure [19]. Tada et al. [20] detected the peak illumination changes in a light source embedded in a deformable structure. Puangmali et al. [21] utilized an optical sensing scheme and presented a three-axis force sensor. Polygerinos et al. [22,23,24,25] also utilized an optical sensing scheme and presented several types of force sensors for catheterization. Liu et al. [26] identified tissue abnormality by a wheeled probe. Ahmadi et al. [27] developed an MRI-compatible optical fiber tactile sensor. Xie et al. [28] presented an optical tactile probe head for palpation. Tan et al. [29] utilized intensity modulation of optical fibers for constructing an MRI-compatible force sensor. By utilizing optical fibers, Su et al. [30,31,32] developed force/torque sensors for prostate needle placement and needle insertion. Turkseven and Ueda [33] also utilized an optical fiber for sensing and feeding back the force information in robotic surgeries. Liu et al. [34] developed an optical force sensor based on low-coherence Fabry–Perot interferometry. However, other related (electrical) components are required to acquire the force information from the transferred signals. This increases the size of the system as a whole and subsequently increases the cost. Magnets and magnetic sensors were also developed [35,36,37]. However, for usage in MRI, no magnetic components are also preferred.

Force visualization is a key for transferring and acquiring the force information without any electrical or magnetic components and with low cost, because the visualized force information can be captured by the eyes of surgeons. Takaki et al. [38] utilized moiré fringe patterns to visualize force information on the forceps. Watanabe et al. [39,40] visualized a small force magnitude by utilizing a highly elastic fabric. They also proposed a stiffness sensing system based on the force visualization mechanism [41]. Although they were not for medical usage, Ohka et al. [42] tracked the position and orientation of columnar and conical feelers made of silicone through a camera. Kamiyama et al. [43] estimated the motion of two-layered markers with cameras to obtain the magnitude, distribution, and direction of the load. However, this paper targeted the development of the neurosurgical instrument, which can perform three functions of retraction, suction, and force-sensing, and its target range of the force information and size of the sensing part were different from those at [38,39,40,41,42,43]. A new suitable force visualization mechanism is required.

Our group has developed the first version of the instrument [44]. However, there were drawbacks. The main drawbacks were the large thickness (16 mm) of the retractor and low force-visualized area. The force-visualized area was the surface of the retractor, that can become covered in blood if bleeding occurs, thus not allowing the force information to be visualized. While retracting deep areas, the surface of the retractor cannot be seen and the force information cannot be obtained. The system presented in this article will resolve these issues.

## 3. Force-Sensible Silicone Retractor Based on Force Visualization Utilizing a Liquid

### 3.1. Target Situation

**Figure 2:**
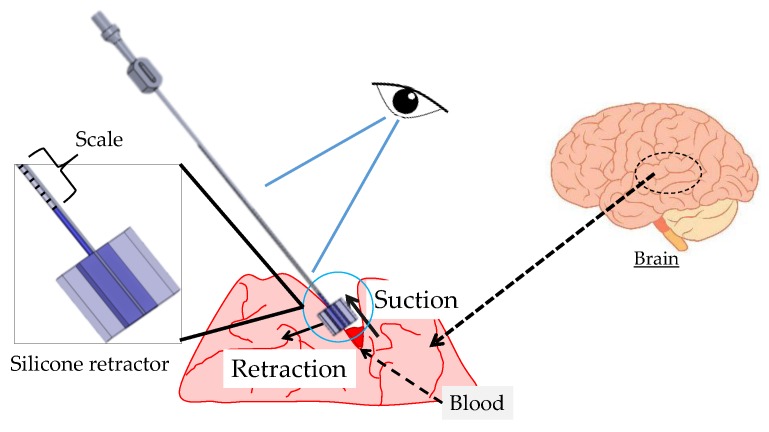
Target situation.

This study proposes a medical instrument with the aim of supporting suction and retraction in neurosurgery. Figure 2 shows the target situation in which the suction pipe removes the fluids using a suction force (for instance, blood in Figure 2). A silicone retractor is attached to the tip of the suction pipe. The silicone retractor has enough width to retract the peripheral tissue. The silicone retractor also has a force sensing component connected to the tube. The level of a liquid inside the tube visually displays the retracting force. Therefore, surgeons can use the suction and retraction functions without changing instruments while observing the retracting load. The retracting force value is displayed on a scale, and since neurosurgeons wear magnifying glasses during the operation; even small marks can be seen. Overload can be then detected by observing the liquid level.

### 3.2. Design Requirements

The design requirements for the proposed silicone retractor are as follows:

1. Suction, retraction, and retracting force measurement are available simultaneously.
2. The silicone retractor is attachable to the suction pipe.
3. There is no electric component in the silicone retractor.
4. The force is visualized.
5. The dimensions should include a width of less than 25 mm, length of less than 15 mm, and thickness of less than 2 mm.
6. The force range should be 0.00–1.00 N, the resolution should be less than 0.05 N, and the minimum sensed force should be 0.10 N.

We realized the three functions by attaching the force-sensible silicone retractor to the suction pipe. The absence of electrical components in the silicone retractor leads to several advantages including easy setup, disposability, easy sterilization/disinfection, MRI compatibility, and low cost.

The force visualization mechanism was used so that surgeons can directly observe the magnitude of the retracting load and avoid an overload on the tissue. The second version of the developed silicone retractor [1] had a length of 16 mm, width of 22 mm, and thickness of 4 mm. In this paper, the requirement for thickness was reduced to 2 mm so that the instrument can be inserted into a narrow space. The requirements for the dimensions were determined according to the suggestions from neurosurgeons. The required range and resolution were the same as the previous system [1] and were based on the studies [13,14,45]. The main objective included avoiding the overload; a resolution of 0.05 N was considered to be sufficiently accurate. At [45], a nominal stress of more than 10 kPa was observed when compressing brain tissue (without breaking). If supposing the contract area of the silicone retractor is 10 mm^2^ (refer to Figure 4), the corresponding force value is 0.10 N, which is the required value for the minimum sensed force.

### 3.3. Principle of Force Sensing

**Figure 3:**
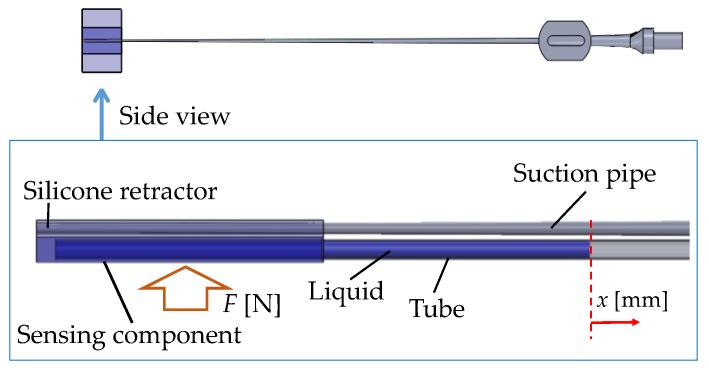
Principle of force sensing.

Figure 3 shows a schematic view of the principle of force sensing. A hole in the sensing component was filled with a liquid (water). The internal portion of the sensor was connected to the tube. The tube was open at one end so that the water can freely move inside the tube. If force is applied at the sensor, it is deformed and the water moves towards the open end of the tube due to its incompressibility. Moreover, owing to the surface tension of the water, its level does not change if the applied force does not change, even if the tube moves. Therefore, the distance travelled by the water is an indicator of the magnitude of the applied force. The diameter of the tubes is small enough for the distance travelled by the water to be captured. Note that this method is valid when the pressure (stiffness of the sensor) is less than or equal to the contact pressure (stiffness of the target object/tissue), as indicated by previous experimental results [46]. Therefore, the system could have a dead zone wherein the water does not move even if the load is applied. The dead zone appears when the load is small. The dead zone is not a problem for avoiding overload if it is far from the value corresponding to overload. Furthermore, the system range and resolution depend on the elasticity of the sensor material.

Let *F* [N] be the magnitude of the retracting force, and *x* [mm] be the moving distance of the edge of the colored water from the initial position. We calibrated the relationship between *F* [N] and *x* [mm] in advance. Next, the load (*F* [N]) is estimated by the displacement of the water (*x* [mm]). To simplify the detection of the displacement (*x* [mm]), colored water was used. By tracking the edge of the colored water, the displacement of the water (*x* [mm]) is determined. The volume of the water did not influence the relationship between *F* [N] and *x* [mm] on account of the incompressibility of the water.

### 3.4. Structure of the Silicone Retractor with an Embedded Force-Sensing System

**Figure 4:**
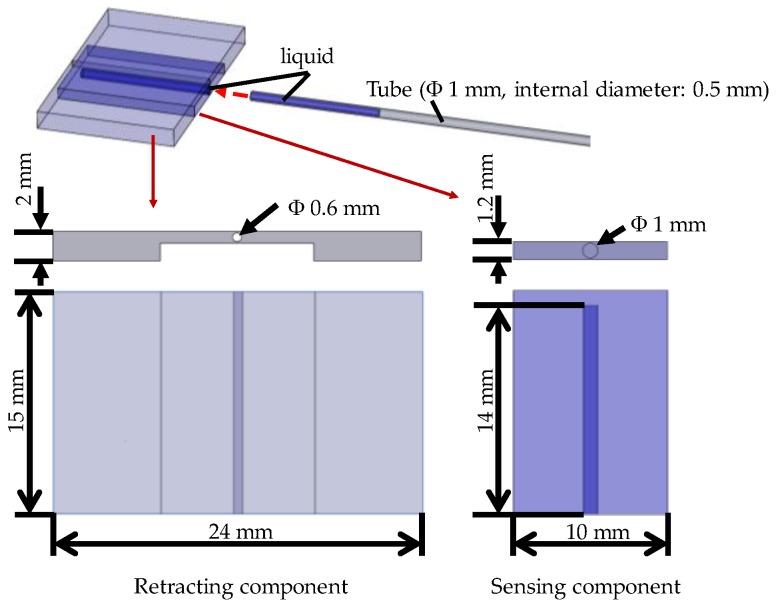
Schematic top and side views of the silicone retractor including a force-sensing component.

Figure 4 shows a schematic view of the structure of the silicone retractor developed in this paper. As shown in Figure 4, the retractor, sensor, colored water, and tube were used to construct the silicone retractor. The retracting component was made of silicone (Liquid silicone: KE-1308, Hardener: CAT1300L-4, Liquid silicone [g]: Hardener [g] = 1:0.06) produced by Shin-Etsu Co., Ltd. (Tokyo, Japan). The sensing component was made of another type of silicone (Ecoflex 00-10) produced by Smooth-On, Inc. (Macungie, PA, USA). The water used was physiological saline solution and colored by an edible blue colorant (food coloring) produced by Kyoritsu Foods Co. Inc. (Tokyo, Japan). The tube used was made of polytetrafluoroethylene (PTFE) (TUF-100 series) produced by Chukoh Chemical Industries Ltd. (Tokyo, Japan) and its inner diameter is 0.5 mm while its outer diameter is 1.0 mm. These parts did not include any electrical components and were low cost, disposable, and easy to sterilize/disinfect. The sensing component was located at the center corresponding to the location of the suction pipe, where the largest load is exerted. The dimensions of the sensing component were: Width, 10 mm; length, 15 mm; thickness, 1.2 mm. The dimensions of the developed silicone retractor were: Width, 24 mm; length, 15 mm; thickness, 2 mm. As mentioned earlier, the retractor must be stiff, while the sensing component must be soft in order to achieve high sensitivity. In [1], only one type of material was utilized to compose the silicone retractor. Here, by adapting hybrid soft sensing and hard retracting structures, a very thin silicone retractor with a thickness of 2 mm was developed.

**Figure 5:**
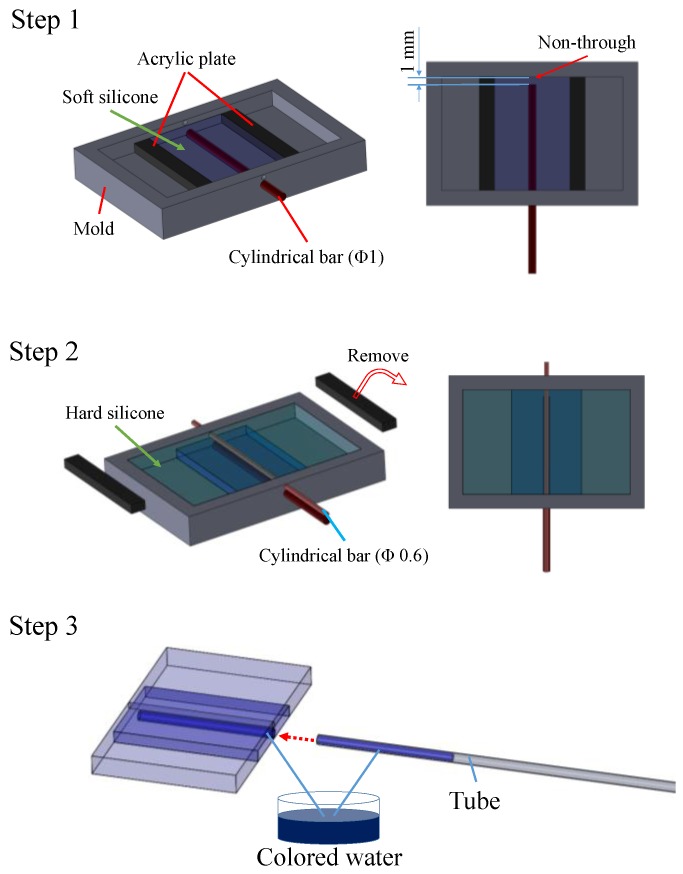
Overview of the manufacturing and assembling processes for the silicone retractor including a force-sensing component.

**Figure 6:**
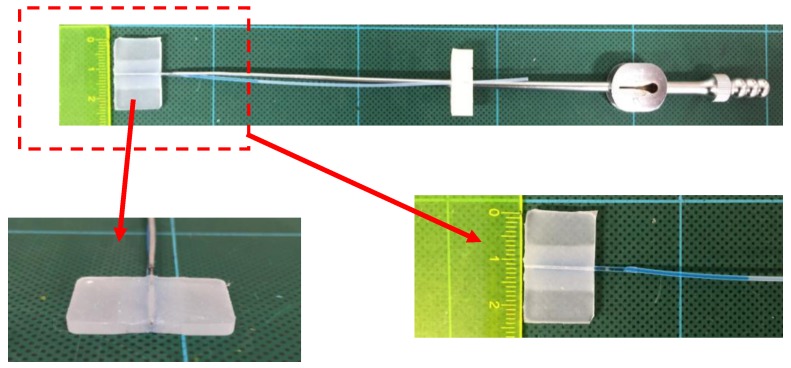
Manufactured silicone retractor including a force-sensing component.

Figure 5 shows an overview of the manufacturing and assembling processes of the force-sensible silicone retractor, which consist of three steps. First, the sensing component is manufactured. Two acrylic plates with a thickness of 1 mm were set on the mold, such that the space separated by the plates has a width of 10 mm and the center of the space corresponds to the center of the mold. Liquid silicone (Ecoflex 00-10, Smooth-On, Inc., Macungie, PA, USA) was poured into the space, and a cylindrical bar with a diameter of 1 mm was inserted into the lower hole to produce the internal portion of the sensor. It should be noted that the bar did not pass through, and was set such that the end of the bar is 1 mm away from the edge, as shown in Figure 5. After the silicone solidified, the acrylic plates were removed; second, the retracting component was fabricated. The liquid silicone used for the retractor was poured into the mold, and a cylindrical bar with a diameter of 0.6 mm was inserted into the upper hole for a suction pipe such that the bar goes through. After the silicone solidified, the two bars were removed, and the solidified silicone was picked up. Note that Ecoflex can easily bond with other types of silicone without requiring any additional materials such as bond. Lastly, the colored water was poured into the hole of the sensor and the tube, and the two were joined (Step 3). We contacted the hole for the sensor with that for the tube, and poured the glue around the contact area to join the holes. This was a prototype, and a bond (CA-522) produced by CEMEDINE Co., Ltd. (Yokohama, Japan) was used to glue the tube into the sensor. A biocompatible bond could also be used. Figure 6 shows the manufactured force-sensible silicone retractor. The retracting and sensing components were made using a monolithic molding.

## 4. Design of the Sensor

### 4.1. Design of the Hole for the Sensor

**Figure 7:**
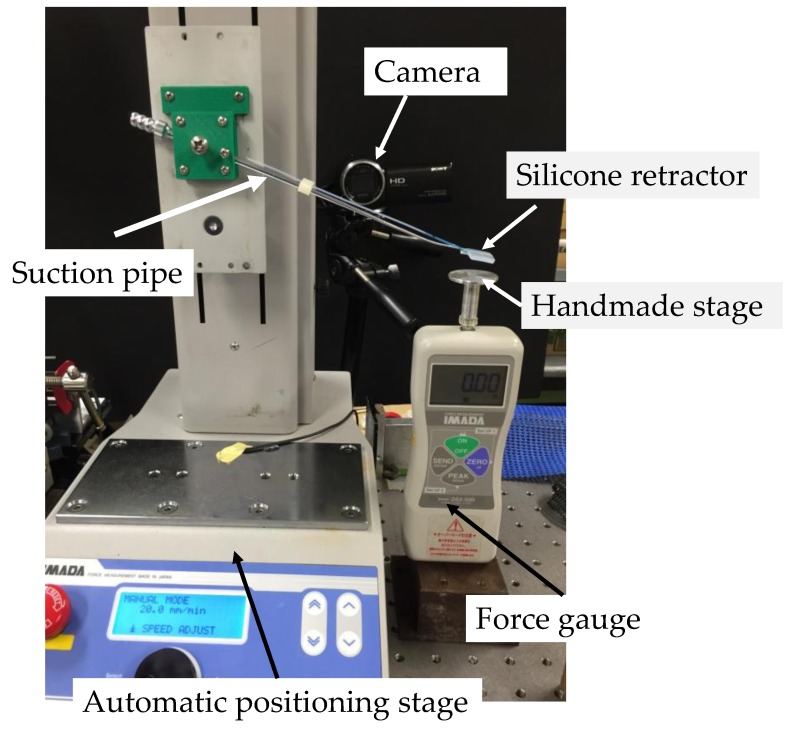
Photograph of the experimental setup.

The structure of the sensing component affects the sensitivity of the sensing system. Here, we investigated the effect of the hole shape on the sensitivity. We conducted experiments in order to investigate the relationship between the liquid displacement and the applied force. Figure 7 shows the overview of the experimental setup. The developed silicone retractor was attached to a suction pipe, which was attached to the automatic positioning stage (MX2-500N, IMADA, Toyohashi, Japan; resolution, 0.01 mm). The force gauge (DS2-50N, IMADA, Toyohashi, Japan) was fixed to measure force in the vertical direction. The handmade stage was placed on the force gauge. A digital camera (HDR-CX675, SONY, Tokyo, Japan; resolution, 1920 × 1080 pixels) was used to capture the displacement of the liquid.

#### 4.1.1. Procedure

**Figure 8:**
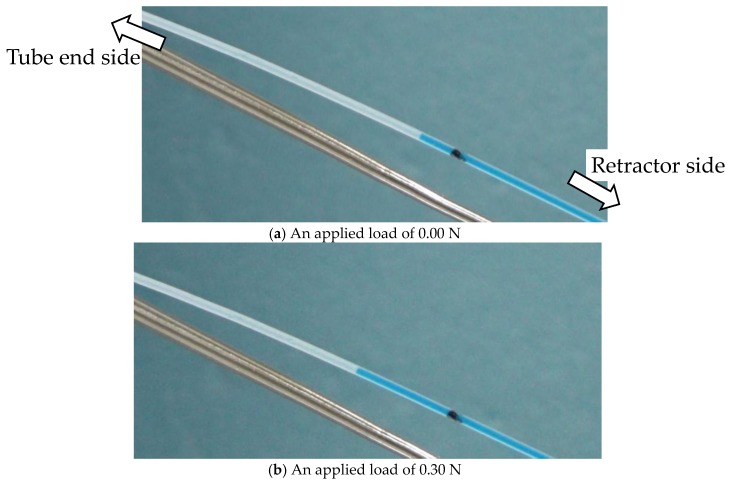
Photograph of the liquid level of the tube when the load was 0.00 N (**a**) and 0.30 N (**b**).

We set such that the sensor of the silicone retractor was contact with the handmade stage on the force gauge. The initial automatic positioning stage was controlled in a way that the value of the force gauge was 0.00 [N]. Next, the automatic positioning stage was controlled to increase the load in increments of 0.05 N until it reached 1.00 N. The speed was 20 mm/min. At each load, a photograph of the colored liquid in the tube was taken to estimate the distance travelled by the edge of the colored liquid. The entire procedure was repeated four times. Figure 8 shows images acquired when the load was 0.00 N and 0.30 N as representative results.

#### 4.1.2. Derivation of the Liquid Displacement

The Photoshop (Adobe, San Jose, CA, USA) software was used to derive the distance moved by the liquid. As shown in Figure 8, a black mark was made on the PTFE tube. Let *x*_0_ be the distance between the mark and the edge of the liquid at 0.00 N, and *x_f_* be the distance at *F* [N]. The distance moved by the liquid was then derived by |*x_f_* − *x*_0_|.

#### 4.1.3. Relationship between the Retracting Force and the Liquid Displacement

**Figure 9:**
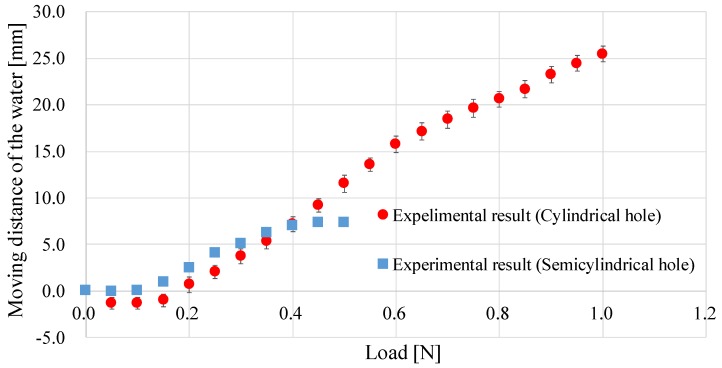
Relationship between the retracting force and the water displacement.

Figure 9 shows the experimental results (red lines with circles and blue lines with squares). When the hole was semi-cylindrical in shape and the applied load was over 0.5 N, the liquid did not move (as there was no liquid inside the sensor) and the results were not shown in Figure 9. In the high load range (0.40–1.00 N), the liquid displacement for the cylindrical hole shape was sensitive enough to capture, while it was hard to capture when the hole was semi-cylindrical in shape. On the other hand, in the low range (0.00–0.40 N), the liquid displacement for the semi-cylindrical hole was more sensitive. The surface of the silicone retractor is originally flat. Upon inserting the suction pipe, it deforms into an inverted V-shape. With increasing the load from 0 N, the silicon retractor goes back to the flat state (original shape) initially, after which the expected deformation (V-shape) is obtained. When the hole is semi-cylindrical in shape, the required load for going back to the flat state is lower than that required for when the hole is cylindrical. Therefore, a higher sensitivity was obtained at a low load range. Note that the negative displacement for the cylindrical hole at a low range (0.00–0.15 N) was due to backflow of the water, resulted from the inverted V-shape. The backflow can be reduced if increasing the size of the hole for the suction pipe, but it is difficult due to the space limitation. In summary, at a high load range, a silicone retractor with a cylindrical hole is preferable; at a low load range, a silicone retractor with a semi-cylindrical hole is recommended, although its sensitivity is not very high. An improvement in the sensor is preferred. Note that we experimentally investigated the effect of a difference in temperature and humidity (humidity is related with capillary force), and did not recognize the effect. It indicates capillary effect at the measurement is negligible.

### 4.2. Improvement of the Sensor Structure

**Figure 10:**
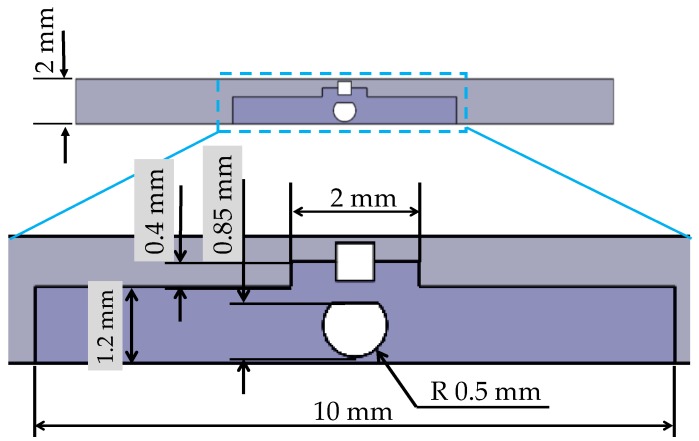
Improved sensor structure.

In order to achieve a high performance in the low range, the sensor structure was improved. As mentioned above, the start of the liquid movement at a low load range (and the reduction of backflow) can be expected if a semi-cylindrical hole is used, while the good sensitivity can be expected over the entire load range if utilizing a cylindrical hole. Therefore, an intermediate shape of the hole as shown in Figure 10 was adopted. Additionally, we increased the soft area around the hole as shown in Figure 10 to achieve better sensitivity. Note that the increase in the soft area more than necessary reduces the retraction performance in the high load range. Therefore, the increase in the soft area was minimized. 

**Figure 11:**
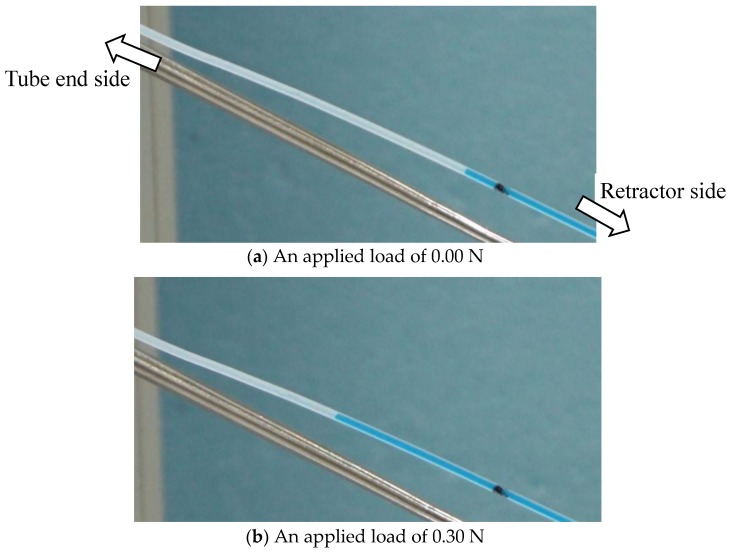
Photograph of the liquid level for the improved silicone retractor at an applied load of 0.00 N (**a**) and 0.30 N (**b**).

**Figure 12:**
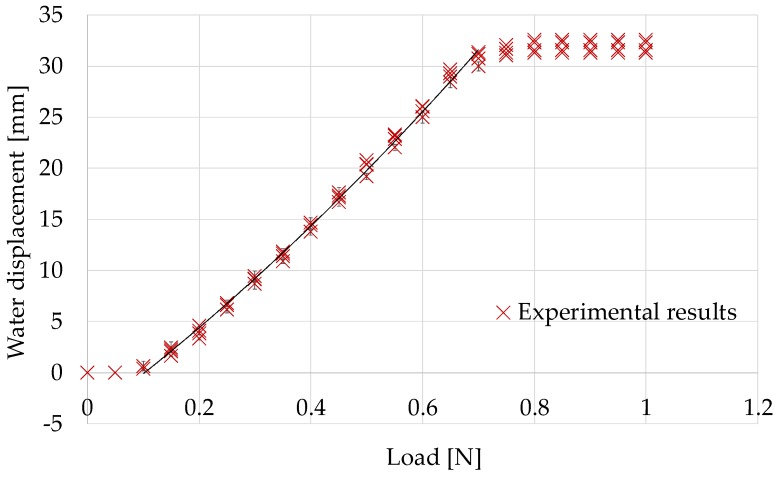
Relationship between the retracting force and the liquid displacement for the improved silicon retractor.

The improved structure was evaluated experimentally. The experimental setup, procedure, and method used to measure the liquid displacement were the same as those used in the previous experiment. The experiment was repeated four times. Figure 11 shows representative images when the applied load was 0.00 N and 0.30 N. Figure 12 shows the experimental results (purple crosses). The line shows the regression curve, which is given by:

F = −0.00011*x*^2^ + 0.022*x* + 0.10 (1)

Table 1 shows the properties of the regression curve, i.e., the obtained coefficient of determination and the root mean squared error (RMSE) between the regression curve and the experimental results. The calculation was performed using the curve fitting toolbox in MATLAB (MathWorks, Natick, MA, USA). The regression curve and RMSE were calculated for the load range of 0.1–0.7 N, because the sensor was not responsive in the other ranges. It is considered that at a load over 0.8 N, there was no water inside the hole of the sensor. It can be observed that the sensing performance was improved. The regression analysis results (Table 1) show that a quadratic function can express the relationship very well. The RMSE value shows that the resolution of the force sensing system was less than 0.05 N (in the range of 0.1–0.7 N).

## 5. Experimental Evaluation on Soft Tissues

**Figure 13:**
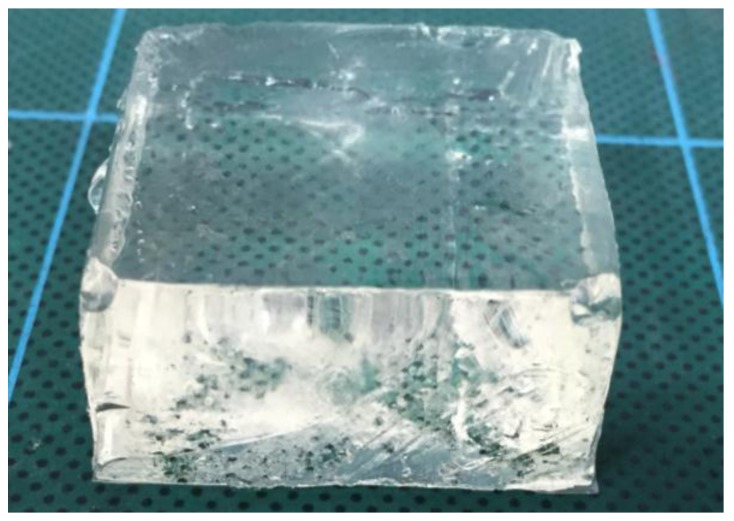
Gelatin used for soft target to simulate brain tissue.

**Figure 14:**
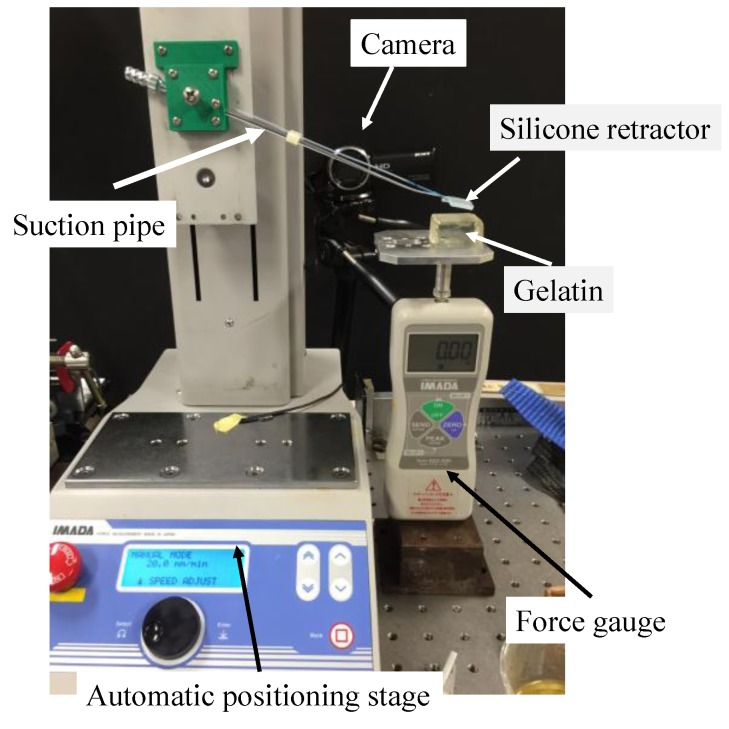
Photograph of the experimental setup while retracting soft surfaces.

In real situations, the target object that is retracted with the silicone retractor is brain tissue (soft objects). Therefore, we investigated the performance of the force-sensing system of the developed silicone retractor when loading gelatin (Figure 13 and Table 2), which resembled the brain tissue. The experimental setup is shown in Figure 14. The experimental setup was the same as that in the previous two experiments, except that the silicone retractor loads the gelatin. The target objects were two kinds of gelatin with different Young’s modulus values. Table 2 shows the dimensions, Young’s modulus, and composition ratio of the two types of gelatin used in the experiment. The Young’s modulus can be controlled by the volume of the gelatin powder. The stiffness of the brain tissue takes various values. For example, the Young’s modulus of cerebral white matter takes the values in the range of 9.3–40.8 kPa [45]. In this paper, Young’s modulus values of 10 kPa and 15 kPa were used as representative examples in order to simulate relatively soft tissue.

**Figure 15:**
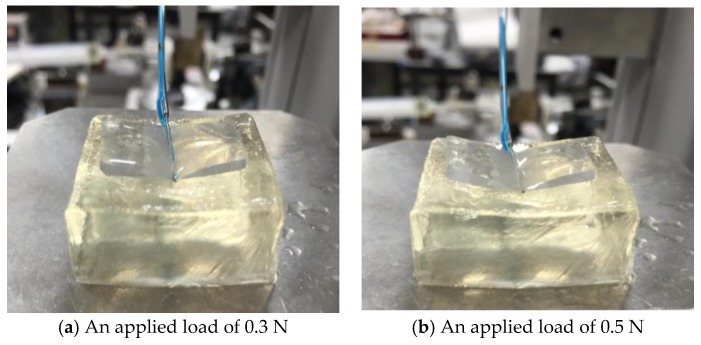
Photograph acquired when retracting the gelatin (Young’s modulus, 15 kPa) with an applied load of 0.3 N (**a**) and 0.5 N (**b**).

The experimental procedure was the same as that used in the previous two experiments, which the only difference being in the range of loading. The range of the applied load was 0.0 [N] to 0.7 [N] to avoid the fracture of gelatin. The experiment was repeated four times. Figure 15 shows representative images captured while retracting the gelatin (Young’s modulus, 15 kPa) when the applied load was 0.30 N and 0.50 N.

### 5.1. Calibration While Retracting Flat and Rigid Surfaces

**Figure 16:**
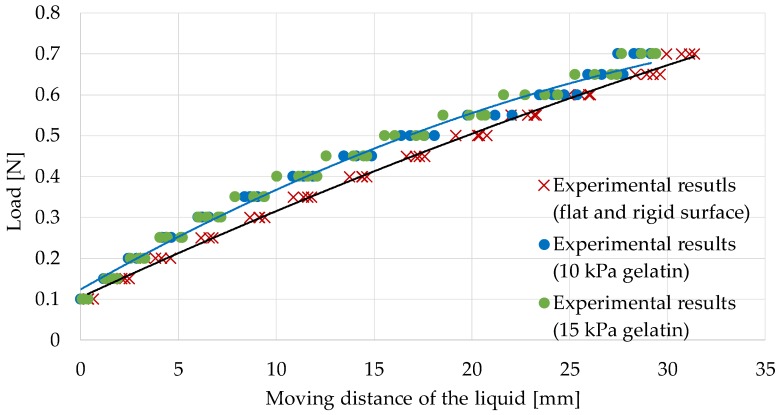
Relationship between the liquid displacement and the retracting force while retracting the two types of gelatin; the black regression curve is provided in Equation (1), and the blue regression curve is provided in Equation (2).

Figure 16 shows the experimental results. The black curve shows the regression curve defined in Equation (1) that was calibrated utilizing the experimental results when retracting flat and rigid surfaces (described in the previous section). Table 3 shows the RMSE between the measured values and those derived from the regression curve (Equation (1)). It is observed that the differences were larger than 0.05 N, but the difference in the RMSE value for the two types of gelatin was very small. When retracting an object, the silicone retractor deformed to a V-shape, as shown in Figure 15. If the target is soft, the deformation becomes is slightly larger than when the target is rigid. This is the reason for the difference. The results suggest that calibration should be done, considering the target stiffness. 

### 5.2. Calibration While Retracting Gelatin (Young’s Modulus, 10 kPa)

With the previous suggestion in mind, we evaluated the experimental results in a way that the calibration and the evaluation targets have similar stiffness levels. Here, we focus on the different regression curve (the blue curve in Figure 16) defined by:

F = −0.00028*x*^2^ + 0.027*x* + 0.12 (2)

Here, the regression curve was derived from the experimental results obtained when retracting gelatin with a Young’s modulus of 10 kPa. Table 4 shows the RMSE between the measured values when retracting gelatin with a Young’s modulus of 15 kPa and those derived from the regression curve (Equation (2)). It can be observed that the regression curve can estimate the load for the gelatin with different stiffness values well. It indicates that the calibration should be performed by utilizing an object with a stiffness level close to that of the target. The results also validated the performance of the force sensing system developed by us.

### 5.3. Evaluation on Curved and Soft Surfaces 

**Figure 17:**
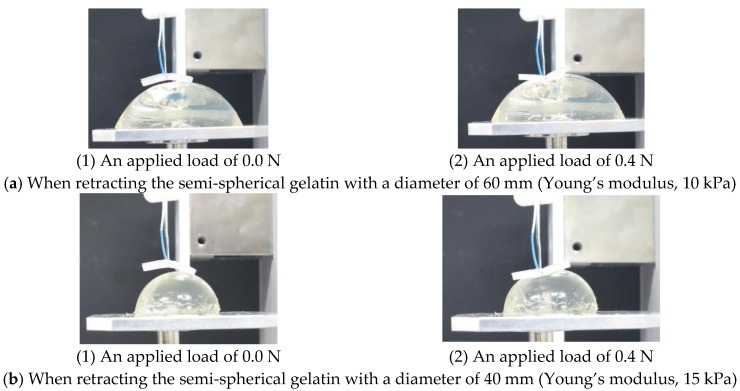
Photograph acquired when retracting the semi-spherical gelatin.

In real situations, the developed system can be used at different angles. Therefore, we investigated the performance of the system when loading semi-spherical gelatin (Figure 17), which simulated the usages at different angles. The experimental setup and procedure are the same as those in the previous experiment, except for the target object and loading point. The target objects were four types of gelatin, whose properties are shown in Table 5. The Young’s modulus was controlled by the volume of the gelatin powder. The loading point was at a distance of 5 mm from the center. The experiment was repeated four times.

Figure 17 shows representative images acquired when retracting the semi-spherical gelatin with a diameter of 60 mm (Young’s modulus, 10 kPa) and the semi-spherical gelatin with a diameter of 40 mm (Young’s modulus, 15 kPa).

**Figure 18:**
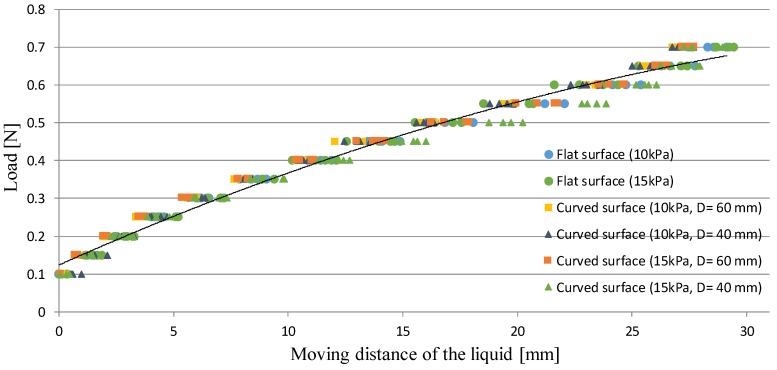
Relationship between the liquid displacement and the retracting force when retracting the four types of semi-spherical gelatin and the two types of flat gelation; the regression curve is provided in Equation (2).

Figure 18 shows the experimental results. The black curve shows the regression curve defined in Equation (2) that was calibrated utilizing the experimental results when retracting the flat gelatin with a Young’s modulus of 10 kPa. Table 6 shows the RMSE between the measured values and those derived from the regression curve (Equation (2)). From Figure 18, the difference was observed at the range of 0.45–0.70 N when retracting the semi-spherical gelatin with a diameter of 40 mm (Young’s modulus, 15 kPa). The actual stiffness is different from Yong’s modulus. If loading a spherical object, the actual stiffness exponentially increases with an increase of loading. If the curvature of the object is high, the increasing rate is high. Therefore, the difference (due to the difference in stiffness) was observed at the range of 0.45–0.70 N when retracting the semi-spherical gelatin with a diameter of 40 mm (Young’s modulus, 15 kPa). Nonetheless, if evaluating RMSE in the separate range, RMSE was 0.018 N at the range of 0.10–0.40 N while RMSE was 0.034 N at the range of 0.45–0.70 N. From the values of RMSE and Table 6, it can be concluded that the resolution was less than 0.05 N. It should be noted that from Table 3, the maximum difference between the actual and estimated forces was less than 0.06 N. Therefore, practically, the retracting force should be estimated while keeping in mind that the resolution is less than 0.06 N even when the stiffness for the evaluation target is far from the one for the calibration target.

## 6. Conclusions

This paper presented a novel neurosurgical instrument, which performs three functions, including retraction, suction, and force sensing. Suction instruments and retractors are frequently used in neurosurgery. To reduce the number of times an instrument is changed and enhance the operation safety, we developed force-sensible silicon retractors, which are attachable to a suction pipe. It was constructed to contain retracting and sensing components. The sensing component contained a hole connected to a tube, and a liquid was placed inside the hole and the tubes. If a load is applied, the sensing component deforms and the liquid inside moves to the end of the tube. Thus, the liquid level corresponds to the retracting force, and the force information can be visually observed. This system is the improved version of our previously developed systems [1,44]. The previous versions had several serious limitations including large thickness, which limited the usability. We resolved this by utilizing a hybrid soft sensing and hard retracting structure, which drastically reduced the thickness of the structure. A medical doctor commented that the developed silicon retractor is able to be inserted into narrow spaces, different from the previously developed ones [1,44]. The comment indicates the improvement of the usability at the proposed system. The force sensing system utilized a force visualization mechanism. Another issue in [44] was that the force-display area was the same as the surface of the silicone retractor, and the force information could not be obtained while inserting the retractor into narrow spaces or deep areas. The usage of a liquid moved the force-display area to around the fingers of the surgeon. The current version of the instrument benefits from the absence of any electrical components, disposability, easy sterilization/disinfection, MRI compatibility, and low cost. We also optimized the structure of the sensor, so that the sensor resolution was less than 0.05 N (0.028 N from Table 6). The sensor range depends on the structure of the sensing component, and we had two types of the ranges (0.2–1.0 N and 0.1–0.7 N). While this is enough to avoid overload, sensing in the low load range (0.0–0.1 N) and accuracy require further improvement.

## Figures and Tables

**Table 1:** Properties of the regression curve.

Dimension of the Polynomial Function	Coefficient of Determination: R^2^	RMSE (Root Mean Squared Error) [N]
2	0.99	0.011

**Table 2:** Properties of gelatin.

Dimension [mm]	Young’s Modulus [kPa]	Composition Ratio
30 × 32 × 16	15	Water:gelatin powder = 100 mL:8 g
30 × 32 × 16	10	Water:gelatin powder = 100 mL:7 g

**Table 3:** RMSE between the measured values and those derived from the regression curve (Equation (1)), which was calibrated utilizing the experimental results when retracting flat and rigid surfaces.

Young’s Modulus [kPa]	RMSE (Root Mean Squared Error) [N]
10	0.051
15	0.054

**Table 4:** RMSE between the measured values when retracting gelatin (Young’s modulus, 15 kPa) and those derived from the regression curve (Equation (2)) calibrated using the experimental results obtained when retracting gelatin with a Young’s modulus of 10 kPa.

Young’s Modulus [kPa]	RMSE (Root Mean Squared Error) [N]
15	0.017

**Table 5:** Properties of semi-spherical gelatin.

Diameter [mm]	Young’s Modulus [kPa]	Composition Ratio
60	15	Water:gelatin powder = 100 mL:8 g
40	15	Water:gelatin powder = 100 mL:8 g
60	10	Water:gelatin powder = 100 mL:7 g
40	10	Water:gelatin powder = 100 mL:7 g

**Table 6:** RMSE between the measured values and those derived from the regression curve (Equation (2)) calibrated utilizing the experimental results when retracting the flat gelatin with a Young’s modulus of 10 kPa.

Diameter [mm]	Young’s Modulus [kPa]	RMSE (Root Mean Squared Error) [N]
60	15	0.026
40	15	0.027
60	10	0.028
40	10	0.026
